# Cross-Cultural Equivalence of De Jong Gierveld Loneliness Scale Among Older Native and Diasporic Chinese Adults

**DOI:** 10.1093/geront/gnaa151

**Published:** 2020-10-13

**Authors:** Sie-Long Cheung, Hans J S M Hobbelen, Cees P van der Schans, Wim P Krijnen

**Affiliations:** 1 Research Group Healthy Ageing, Allied Health Care and Nursing, Hanze University Applied Sciences Groningen, The Netherlands; 2 Department of Health Psychology, University Medical Center Groningen, University of Groningen, The Netherlands; 3 Department of General Practice and Elderly Care Medicine, University Medical Center Groningen, University of Groningen, The Netherlands

**Keywords:** Cross-national comparison, Item response theory, Mental health, Psychometric properties

## Abstract

**Background and Objectives:**

Loneliness is prevalent among older adults and known to be detrimental to mental health. The objective of this study was to determine the psychometric properties of the Chinese 6-item De Jong Gierveld Loneliness Scale (DJGLS) in the older native and diasporic Chinese community.

**Research Design and Methods:**

Participants were recruited from a local community in urban Tianjin, China and urban Chinese communities of older adults in the Netherlands. Scale properties, including reliability, were calculated with Cronbach’s alpha and multiple-group confirmatory factor analysis to examine the 2-dimensional structure of the scale and the cross-cultural equivalence between both countries. Item response analysis was employed to plot the relationships between the item response and expected total scale score.

**Results:**

A total of 193 older adults from China and 135 older adults from the Netherlands were included. The Cronbach’s alphas were 0.68 (China) and 0.71 (the Netherlands). The DJGLS’s 2-dimensional structure was validated by the goodness of fit and the factor loadings. Cross-cultural equivalence was demonstrated with the multiple-group confirmatory analysis. In addition, sufficient discriminative power of the individual items was demonstrated by item response analysis in both countries.

**Discussion and Implications:**

This study is the first to provide a detailed item behavior analysis with an item response analysis of the DJGLS. In conclusion, the findings of this study suggest that the DJGLS has an adequate and similar item and scalar equivalence for use in Chinese populations.

Human beings have a natural tendency to be socially involved with others, and feelings of loneliness are undesirable. Belonging and social involvement, therefore, are fundamentally important to individual well-being (Baumeister & Leary, 1995). Weiss (1973) conceptualized loneliness as consisting of emotional and social loneliness. Emotional loneliness refers to a feeling of lacking intimate relationships, whereas social loneliness implies a feeling of lacking social integration and embeddedness (Weiss, 1973). As they age, older individuals encounter negative experiences such as the loss of a spouse, friends, or family members and a decline in health that impedes social engagements and intimate relationships. Loneliness is generally believed to be a serious problem for older populations such as the older Chinese diaspora in Western countries. They must bridge cultural distances in their host country due to the shift of once being a native member in their country of origin to become a minority in their new environment, and this may be an additional contributing factor to social vulnerabilities such as loneliness.

## Loneliness in Older Chinese Populations

Worldwide, the ethnic Chinese constitute the largest aging population (Health and Places Initiative, 2015; Tseng et al., 1995). The accumulated literature focusing on loneliness among the older native Chinese adults and, more recently, among aging Chinese immigrants in Western societies reflects a growing concern for this population. Loneliness among older adults is prevalent among 15%–30% of the older adult population in China and 20% of that in Hong Kong (Leung et al., 2008; Luo & Waite, 2014; Yang & Victor, 2008). In the older adult Chinese community in Chicago, loneliness showed a prevalence of 26% (Simon et al., 2014). Moreover, there is a higher prevalence of loneliness among older Chinese adult immigrants than among the older native adult population in Australia, the United Kingdom, and Canada (Ip et al., 2007; Victor et al., 2012; Wu & Penning, 2015). However, as stressed by Yang and Victor (2008), cross-national comparisons of loneliness measures remain tentative unless loneliness instruments have demonstrated to have generalizable measurement properties.

## Loneliness Measurement Instruments

Two scales that are frequently used to measure loneliness are the UCLA Loneliness Scale (Russel, 1996) and the De Jong Gierveld Loneliness Scale (DJGLS; De Jong Gierveld & Kamphuls, 1985; De Jong Gierveld & Van Tilburg, 1999). Whereas the former is unidimensional, the DJGLS is based on a two-dimensional structure of loneliness as conceptualized by Weiss (1973) comprising social and emotional loneliness. Since the original 11-item DJGLS was developed, a shorter version, the 6-item DJGLS, has been constructed (De Jong Gierveld & Van Tilburg, 2010). The latter was developed to facilitate the measurement of loneliness by preventing response fatigue, making it more suitable for inclusion in larger surveys, especially those considering multiple measurements.

## Cross-Cultural Equivalence of Measurements

Validation of measurements’ cross-cultural equivalence is crucial as the lack of such could lead to invalid and biased results and, therefore, yield misguiding conclusions. Well-established scholarly works provide theoretical underpinnings of cross-cultural validation. Herdman et al. (1997) describe different schools of thought concerning cross-cultural research: absolutism, universalism, and relativism. The absolutist approach believes that there is a trivial influence of culture on measurements and thus these have universal equivalence by definition. Universalism implies that culture has a significant influence on conceptual understanding. Validation methods, therefore, are required to achieve cross-cultural equivalence. Relativism assumes that the substantial influence of culture inhibits measurement instruments to achieve cross-cultural equivalence. In this study, the universalist approach is taken toward the cross-cultural validity of loneliness measurements.

The framework of Hui and Triandis (1985) provides guidance for validating cross-cultural equivalence of measurements. Four categories of equivalence are organized based on an abstract–concrete continuum of equivalence: conceptual/functional, operationalization, item, and scalar. Concept/function equivalence is the most abstract form and requires a construct to be of similar relevance in different cultures. Operationalization equivalence is attained when the mode of measurement does not affect the outcome; a rigorous translation methodology would contribute to this level of validation. The conceptual/functional and operationalization equivalence are overlapping. Meaning, inadequate translations of scales would lead to loss of concept equivalence, which would consequently result in irrelevant items that are of little significance to the target population. Thus, translation, for example, could contribute to both concept and operationalization equivalence. The more concrete levels of equivalence refer to the item and scalar equivalence, respectively. Item equivalence resembles the relevance of the instrument’s items to the latent trait, and the item response theory (IRT) provides a framework for such an investigation. Scalar equivalence regards psychometric properties such as the well-known factor analyses, among others.

## Advancing the Chinese Version of De Jong Gierveld Loneliness Scale

The DJGLS has been translated into several languages, including Chinese, and cross-culturally validated in several countries. The Chinese version of the six-item DJGLS has been translated and partially psychometrically validated in the older Chinese adult population in Hong Kong (Leung et al., 2008). Two categories of equivalence have been previously comprehensively demonstrated in the literature. First, the concept equivalence of loneliness is implied by its sound theoretical foundation, and existing research discussing it among older Chinese native populations and diaspora suggests a universality of the concept as proposed by Weiss (Bennet et al., 2018; Dong et al., 2012a; Li et al., 2017; Morgan et al., 2020; Wang et al., 2011; Weiss, 1973). Second, the development of the Chinese version of the short DJGLS by Leung et al. (2008) provided operationalization equivalence by rigorous translation procedures involving Delphi panel ratings. Moreover, scalar equivalence was partially determined by assessing its content validity. However, extensive data on the item and scalar equivalence of the instrument remain lacking, such as construct validity and item response analysis. The cross-cultural validity of the Chinese version of DJGLS, therefore, could benefit from additional examination in its item and scalar equivalence.

Although there are a substantial number of commonalities that can categorize all ethnic Chinese as one cultural group, the ethnic Chinese can be categorized into many subgroups with their distinct characteristics, such as age or gender. However, the diaspora can also be perceived as a distinct subgroup within the larger ethnic Chinese population. First, literature has provided possible entries for a nuanced image of the older Chinese diasporic population due to long-term exposure to Western adults in the United States perceive that loneliness is determined by the lack of harmonious intergenerational relationships which, similar to native Chinese populations, is rooted in the cultural beliefs of Confucianism. Despite that the diaspora regards Confucian values as important, these values are prone to modification, if not erosion, in diasporic communities in Western societies (Dong et al., 2012b; Hsueh & Clarke-Ekong, 2008; Lan, 2002; Liu et al., 2000; Lo & Russell, 2007). Therefore, the Chinese diaspora can be considered as a social construct, meaning its integration in Western society leads to the formation of a hybrid identity and a diasporic culture that diversifies the contemporary Chinese culture (McKeown, 1999).

Second, the diasporic community has a different demographic profile than the mainland Chinese community. The ethnic Chinese also include those who have ancestral roots in China but are part of a multigenerational migrant family. For example, while they are officially recognized as Malaysian or Singaporean because of their birth country, they are, in fact, ethnically and culturally part of the larger Chinese population. According to Vandenberg and Lance (2000), it is required that measurement equivalence be demonstrated within different subgroups from one cultural group before cross-cultural equivalence is achieved. This could especially be the case for the Chinese population as the Chinese culture comprises a variety of beliefs and attitudes.

This makes the Chinese diaspora a diverse population. Following, it can be reasoned that the cross-cultural equivalence of instruments that are validated in Chinese societies need additional examination for the diaspora. Thus, there is a need to investigate whether the DJGLS attains a comparable validity among the Chinese diaspora and the native Chinese. Furthermore, an evaluation of measurements across subgroups over countries would attribute to the degree of their generalizability (Prince, 2008).

Therefore, this study aims to contribute to the existing body of knowledge by (a) providing additional insights in the item and scalar equivalence of the Chinese version six-item DJGLS, (b) demonstrating the use of item response analysis as a sophisticated validation method for item equivalence, and (c) investigating possible differences in the validity of the DJGLS between the older native and diasporic Chinese community in China and the Netherlands.

## Method

### Study Samples

Older Chinese adults from two populations were included in this study: community-dwelling older adults living in the Netherlands and in urban Tianjin, China. Those from the Netherlands were individuals who considered themselves to be Chinese. These included Chinese migrants from countries other than China or Hong Kong. The older Chinese adults in Tianjin included urban community-dwelling persons. There was no distinction made based on whether they were “locally raised” in Tianjin. Birth country was not the main indicator but, rather, the spoken language (either a Chinese language or dialect).

### Participant Recruitment and Data Collection

#### China

Older adults in urban Tianjin were personally invited by students who approached them while the older adults were engaging in leisure activities in their local neighborhoods. They were asked to participate in the study and to complete the questionnaires that were all self-reporting. As the main language was Mandarin, the version with formal Chinese translation was used. The data were aggregated from April to May 2019.

#### The Netherlands

Participants were recruited from several urban regions. Older Chinese adults were invited to participate in the study through a national older Chinese adult association. During the local meetings of the community, the first author introduced the study to the members. The data were collected from November 2018 to March 2019. The main language within this community was Cantonese, and therefore, the participants were provided with the choice between the Cantonese and formal Chinese translations of the DJGLS. In 10% of the cases, the form was completed by an acquaintance of the respondent due to illiteracy. Data from the illiterate respondents were treated in an equal manner as those of the literate respondents in the analysis as it had a minimal influence on the data.

#### Chinese version of the six-item DJGLS

The Chinese short version of the DJGLS was used as developed by Leung et al. (2008). It includes formal Chinese and additional Cantonese translations that are specifically tailored to the Cantonese-speaking population. Both versions have been previously validated for equivalence by a bilingual expert panel (Leung et al., 2008).

Items 1–3 are negatively phrased whereas items 4–6 are positively phrased (De Jong Gierveld & Van Tilburg, 1999). For the negatively phrased items, the scoring was “Yes” (3 points), “More or Less” (2 points), and “No” (1 point); points were reverse-scored for positively phrased items. To define the loneliness categories, the manual for the DJGLS was used (De Jong Gierveld & Van Tilburg, 1999).

For the psychometric analyses, a scoring method that was different from the manual was employed; the scores of items 4–6 were reversed, and the sum of all scores was used. After this, an increased score indicated a higher degree of reported loneliness in all of the cases. The sum of the item scores has a minimum of 6 and a maximum of 18; the frequency of these scores, respectively, corresponds to floor and ceiling effects.

### Statistical Analysis

All data were analyzed using R version 3.6.0 (R Core Team, 2013). Cases with four or more missing answers were omitted. In the event of missing values, the sample mean rounded to the nearest integer was substituted.

The reliability of the scale was determined by Cronbach’s α where a value larger than 0.70 was considered as a satisfactory result (Nunnally, 1978). The item behavior of the scale was investigated by the use of the polyserial correlation coefficient using item response analysis with the KernSmoothIRT library (Mazza et al., 2014) and Psych library (Revelle, 2018). IRT was used in the analysis as it provides a framework for sophisticated prediction of the latent response per item (Embretson & Reise, 2013). Specifically, the “Kernel Smoothing IRT” was utilized to investigate the relationship between a latent factor and the score per item with a polyserial correlation coefficient that was used to calculate the relationship between each item and the latent factor (Mazza et al., 2014). A higher coefficient indicated a stronger relationship. Item-factor dependencies were visualized with plots of items with the expected latent scores on each of the loneliness factors. A higher score on the latent trait (“expected score” on loneliness) gives a higher item score (“expected item score”).

The two-dimensional structure of the DJGLS was examined with multiple-group confirmatory factor analysis (MGCFA). With MGCFA, the correlation between latent variables (social and emotional loneliness) and corresponding items was estimated by factor correlations and item loadings, respectively. Additionally, the similarity between the factor models for the native and diasporic Chinese community was tested to investigate cross-cultural invariance. As the MGCFA was performed on items, a robust version was carried out with the Satorra–Bentler correction (Satorra & Bentler, 2010). The MGCFA was performed with the “lavaan” library in R (Rosseel, 2012). A *p* value of less than .05 was considered statistically significant.

## Ethical Procedures

Permission for the study was granted by the Medical Ethical Committee from the University Medical Center Groningen and Tianjin University of Traditional Chinese Medicine. Respondents were informed prior to their participation about the study purpose, the consequences of the provided information, and their right to withdraw at any time. Permission to use the scale was obtained from the original authors (Leung et al., 2008).

## Results

### Demographic Background

The study sample from China included 193 participants and that from the Netherlands included 135 participants after the deletion of a small number of cases with missing responses (China *n* = 6; the Netherlands *n* = 18). The demographic background characteristics are listed in Table 1. The sample from China included those who were mostly locally born in Tianjin (67%) and predominantly cohabiting (82%) and married (83%). The sample in the Netherlands predominantly consisted of older Chinese adults born in China (43%) and Hong Kong (42%), many of whom were cohabiting (69%) or married (66%).

**Table 1.** Study Sample Characteristics

	China	The Netherlands
Study sample (*n*)	193	135
Age (in years)		
Mean (*SD*)	70.3 (6.5)	70.7 (8.8)
Gender (female, %)	53	59
Years lived in the Netherlands/Tianjin		
Mean (*SD*)	51.6 (26.7)	39.3 (9.8)
Country of birth (%)		
China	100	43
Hong Kong	0	42
Malaysia	0	5.2
Indonesia	0	1.5
Surinam	0	0.7
Singapore	0	3.7
Taiwan	0	1.5
Vietnam	0	0.7
Missing	0	1.5
City of birth (%)		
Tianjin	67	—
Outside Tianjin	33	—
Education level (%)		
No education	5.0	9.2
Elementary school	15	35
Middle school	38	40
High school	26	10
Bachelor’s or higher	17	3.9
Missing	0.0	2.0
Living situation (%)		
Alone	19	28
Missing	0.0	3.0
Marital status (%)		
Unmarried	1.5	3.7
Married	83	66
Divorced	1.5	13
Widowed	14	16
Missing	0.0	2.2
Loneliness score (%)		
Not lonely	52	25
Moderately lonely	42	47
Severely lonely	6.2	27
Ceiling effect (%)	0.5	1.5
Floor effect (%)	30	9.6

### Reliability

According to Cronbach’s α, the reliability of the emotional loneliness subscale was 0.61 (CI: 0.53–0.69) and 0.59 (CI: 0.46–0.71) and that of social loneliness was 0.83 (CI: 0.78–0.87) and 0.81 (CI: 0.76–0.87) in China and the Netherlands, respectively. The DJGLS as a whole had a reliability of 0.68 (CI: 0.60–0.75) in China and 0.71 (CI: 0.63–0.79) in the Netherlands, as summarized in Table 2.

**Table 2.** Cronbach’s α If One Item Is Deleted per Subscale, for the Subscale and Total Scale (90% Confidence Intervals); Point Polyserial Correlation Coefficients Between Loneliness Factor and Each Item

	China	The Netherlands	China	The Netherlands
	Cronbach’s α (if an item is deleted per subscale)	Cronbach’s α (if an item is deleted per subscale, emotional and social subscale, and the total scale)	Polyserial coefficient	Polyserial coefficient
*n*	193	135	193	135
Emotional loneliness	0.61 (0.53–0.69)	0.59 (0.46–0.71)		
1. I experience a general sense of emptiness	0.40	0.40	0.65	0.63
2. I miss having people around	0.42	0.60	0.63	0.47
3. I often feel rejected	0.63	0.47	0.41	0.65
Social loneliness	0.83 (0.78–0.87)	0.81 (0.76–0.87)		
4. There are plenty of people I can rely on when I have problems	0.85	0.80	0.66	0.70
5. There are many people I can trust completely	0.68	0.71	0.70	0.70
6. There are enough people I feel close to	0.76	0.72	0.67	0.72
Loneliness	0.68 (0.60–0.75)	0.71 (0.63–0.79)		

### Construct Validity

#### Multiple-group confirmatory factor analysis

The factor loadings for the MGCFA confirmed the hypothesis of having two factors in both countries, as presented in Table 3. All items demonstrated a relationship with the respective latent factor as all item loadings were significantly positive (*p* < .001). Item 3 (“I often feel rejected”) showed a lower loading than the other items in China. All item loadings in the Netherlands were similar.

**Table 3.** Multiple-Group Confirmatory Factor Analysis’ Factor Loadings (Standard Errors), Factor Correlations and Goodness-of-Fit Indices, and Pearson Product-Moment Correlations

	China	China	The Netherlands	The Netherlands
	Emotional loneliness Factor I	Social loneliness Factor II	Emotional loneliness Factor I	Social loneliness Factor II
*n*	193	193	135	135
Emotional loneliness				
1. I experience a general sense of emptiness	0.52* (0.08)	—	0.46* (0.08)	—
2. I miss having people around	0.55* (0.08)	—	0.31* (0.09)	—
3. I often feel rejected	0.19* (0.05)	—	0.45* (0.08)	—
Social loneliness				
4. There are plenty of people I can rely on when I have problems	—	0.44* (0.06)	—	0.50* (0.06)
5. There are many people I can trust completely	—	0.56* (0.05)	—	0.58* (0.05)
6. There are enough people I feel close to	—	0.48* (0.06)	—	0.59* (0.05)
Correlation Factors I and II	0.26**		0.37**	
χ ^2^	7		8.8	
*df*	16		16	
Robust RMSEA (<0.08)	0.000		0.036	
SRMR (<0.08)	0.027		0.042	
Robust CFI (≥0.90)	1.000		0.992	
Pearson product-moment correlation***	0.957*		0.967*	

*Note:* χ ^2^ = chi-square model; CFI = comparative fit index; *df* = degrees of freedom; RMSEA = root mean square error of approximation; SRMR = standardized root mean square residual.

*p ≤ .001, **p ≤ .01, ***p ≤ .05.

To investigate the invariance of the items, three MGCFA models were estimated and compared as summarized in Table 4. Model 1 has no constraints with all of its parameters estimated separately for both countries. Model 2 has all its loadings constrained to be equal between both countries. Model 3 equals Model 2 except that the loadings for item 3 are not constrained to be equal. Model 1 shows a good fit with a comparative fit index (CFI) of 1.000 and root mean square error of approximation (RMSEA) of 0.000 (Hu & Bentler, 1999; Kline, 2015). The fit indices remain similar between the three models that are hierarchically related and tested with the likelihood ratio test. When comparing Models 1 and 2, there is a significance of *p* = .03, meaning that both models are different. A comparison between Models 1 and 3 gave a *p* value of .3, meaning that these models were not different. These data provide evidence for the invariance between both countries, except for Item 3 (Table 4).

**Table 4.** Goodness-of-Fit Indices of Multiple-Group Confirmatory Factor Analysis and Model Testing Between Models 1 and 2, and Models 1 and 3

	AIC	BIC	CFI	RMSEA	χ ^2^ (*df*)	Δχ ^2^ (Δ*df*) (compared with Model 1)	*p* (compared with Model 1)
Model 1: No constraints imposed	3,545.3	3,689.5	1.000	0.000	18 (16)	—	—
Model 2: Invariant factor loadings	3,550.9	3,672.2	0.997	0.053	36 (22)	14 (6)	.03
Model 3: Invariant factor loadings except Item 3	3,542.7	3,667.9	0.997	0.031	26 (21)	7.4 (5)	.3

*Note:* AIC = Akaike information criterion; BIC = Bayesian information criterion; CFI = comparative fit index; RMSEA = root mean square of approximation.

**Figure 1.**
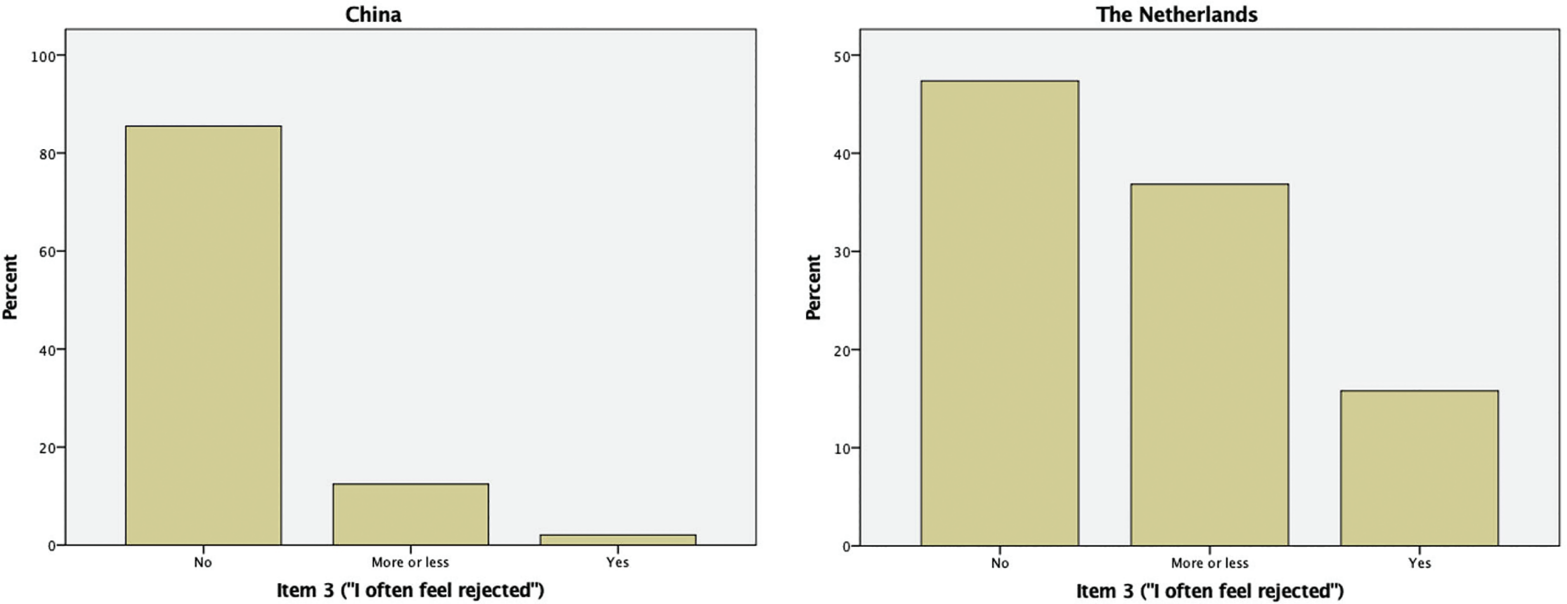
Response pattern of Item 3 (“I often feel rejected”) in China and the Netherlands.

As Item 3 demonstrated a much lower item loading for China in the MGCFA, the data showed a different data distribution pattern between both countries as presented in Figure 1. In China, 86% of the sample scored “no” to “feeling rejected” contrary to 47% in the Netherlands.

#### Pearson product-moment correlation

The association of the total subject score and latent factor was explored with Pearson’s product-moment correlation. This provided a high correlation with an *r* = 0.957 ± 0.003 for the sample in China and *r* = 0.969 ± 0.009 for the sample in the Netherlands.

### Item Response Analysis

**Figure 2.**
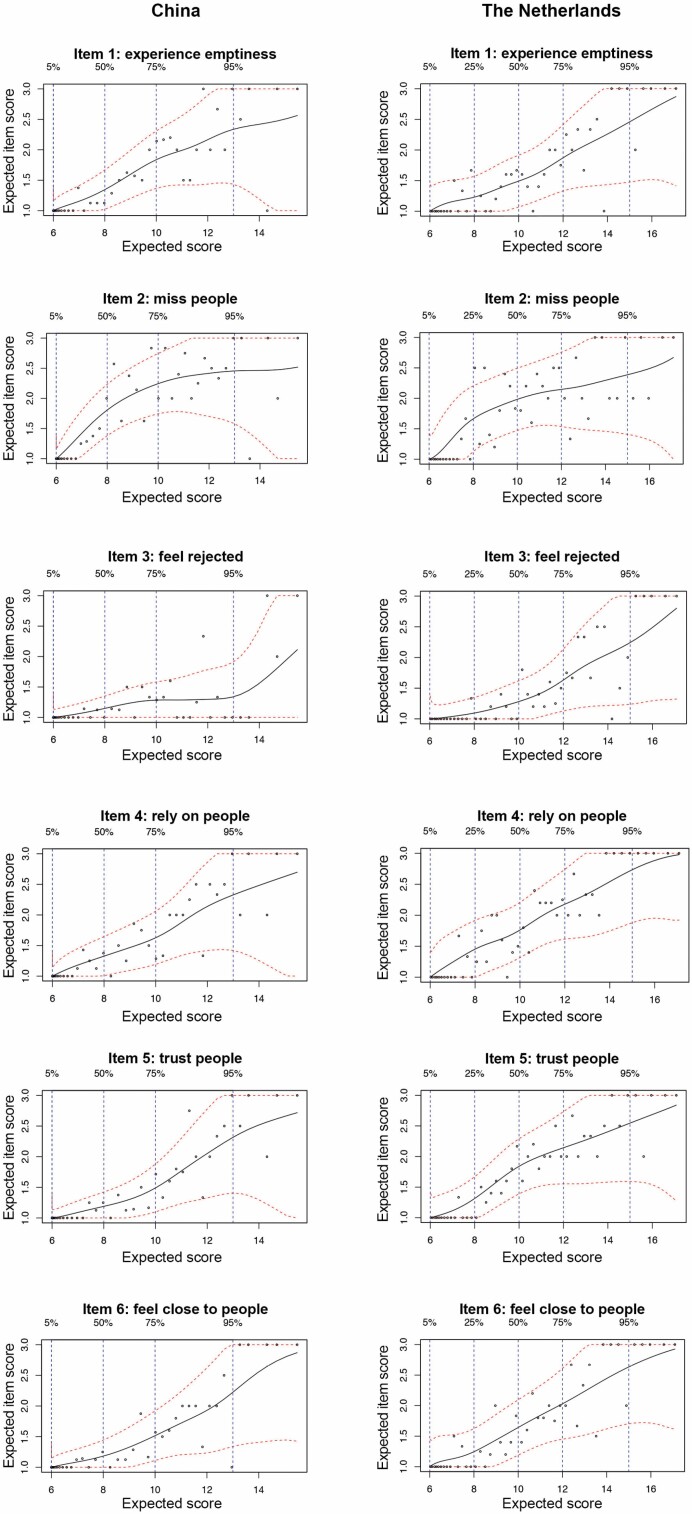
Expected loneliness and expected item scores for all subjects with kernel smoothly fitted curves in black and their 95% confidence intervals in red for each item from the study samples in China to the left and from the Netherlands to the right.

The polyserial correlation coefficients between each of the latent loneliness factors are presented in Table 2. The item characteristic curves depict a positive relationship throughout the scoring range on the scale in both countries, as shown in Figure 2. In all of the cases, the models indicate that an increase in the expected loneliness score corresponds with an increase in the expected item score, except for Item 3 regarding “feeling rejected” in the sample in China. The latter shows a relatively weak relationship between the item score and the expected loneliness score. For the majority of the scoring range (loneliness scores between 6 and 14), the expected score for the feeling rejected item is 1 point (meaning “no feelings of rejection”) until the extremely high end of “severe loneliness” for which the expected item score is 2 (“more or less having feelings of rejection”).

## Discussion

This study evaluated the item and scalar equivalence of the Chinese version of the six-item DJGLS among Chinese older adults in the Netherlands and in China. The results suggest that the instrument is adequate and has similar psychometric scale properties and item properties in both countries. Specifically, Pearson’s product-moment correlation and MGCFA showed that the item loadings and correlation coefficients are similar between the two countries, except for the third item of the scale. This indicates that the Chinese version of the DJGLS is sufficient for use for the Chinese population. Overall, reliability of the subscales was sufficient as only the emotional loneliness subscale was significantly below the norm in China. All in all, despite the differences between the older Chinese adults living in China and the diaspora in Western society, the scale largely demonstrated similar results, and the current research found evidence for its cross-cultural equivalence.

The scale is a beneficial addition for research on loneliness, as noted by the original authors (De Jong Gierveld & Van Tilburg, 1999). The study of loneliness among the older international Chinese diasporic populations in Western countries, such as European countries and the United States as well as in China, can be well measured with the use of the six-item scale with accurate cross-national data comparisons. This provides ample opportunities in international comparison research to study the older generation in various settings while measuring the shared universal feelings of loneliness and the distinctions between social and emotional loneliness.

Although the item loadings of the factor analysis demonstrated insights into the items’ relationship with the latent factor, it describes the items in a single parameter. The positive loadings demonstrate that larger item scores tend to occur with larger loneliness latent trait scores. However, the current IRT analysis provides a more detailed analysis than the MGCFA on the item’s discriminative power throughout all degrees of loneliness (from “no loneliness” to “severe loneliness”). In this study, the data show that the items are related to the latent factor, loneliness, in both China and the Netherlands. Although the confidence intervals in the plots are relatively large due to the small sample sizes, the results are consistent with the item loadings of the MGCFA; that is, items with higher factor loadings demonstrated stronger discriminative power in the IRT analysis.

Item 3 (“feeling rejected”) showed a low factor loading on the MGCFA in China and corresponded with the IRT analysis as the same item showed weak discriminative power. Consistent with the literature, the same item has one of the lowest item loadings in the factor analyses of the original 11-item DJGLS (Çavdar et al., 2015; Iecovich, 2013; Uysal-Bozkir et al., 2017). It is possible that the IRT analysis of Item 3 reflects the strongly uneven distribution of the “feeling rejected” item, and thus loneliness in the study sample of China, as the current IRT analysis is a more sensitive approach. This hypothesis is confirmed by the study of Buz and Pérez-Arechaederra (2014), where it is shown that the “feeling rejected” item is one the most difficult items, meaning that it is particularly highly accurate in discriminating the higher extreme levels of loneliness.

Similar to this study, Leung et al. (2008) found that 94% of the native Chinese respondents reported nonaffirmative to “feeling rejected.” Moreover, this study shows that the diasporic sample scored relatively high on the “feeling rejected” item. These findings can be considered in the light of previous literature where non-Western immigrants in Europe experience discrimination, which could have resulted in “feeling rejected” on the DJGLS (Brüß, 2008; Cook, 2010; Kunuroglu et al., 2018; Palmberger, 2017). This could explain the disparate response patterns regarding the “feeling rejected” item between the native and diasporic respondents. Whether this difference persists over other diasporic communities should be confirmed by additional comparative research between diasporic and native Chinese communities. Furthermore, it can be derived from the literature that the native population only scores positively on “feeling rejected” in cases of extreme loneliness.

On the basis of these reasons, it is expected that the “feeling rejected” item demonstrates weak discriminative loneliness properties in the specific study sample in China on the IRT analysis. This could also be an explanation for the “feeling rejected” item having good discriminative properties in the case of the Netherlands, as the study sample gave a normal distribution from “no loneliness” to “severe loneliness.” Nonetheless, this finding does not undermine the six-item Chinese version DJGLS as a whole, as the combination of the six items provides sufficient discriminative power to measure loneliness. Therefore, the IRT not only shows the discriminative power of the individual items but also provides insight into how different items can complement each other with their discriminative capacities within the scale. In addition, the IRT analysis also demonstrated the expected positive relationship between each item score and the loneliness score.

One limitation of this study is that the study sample in the Netherlands had more illiteracy than the study sample in China, mainly among women 80 years and older. The study sample in China managed to complete the questionnaires by autonomous self-report. This is also reflected in the education level; the study sample in China reported a higher degree of education, whereas the older Chinese adults in the Netherlands generally had attained relatively lower levels of education. For illiterate individuals, the questionnaire was completed with the help of an acquaintance, which may have resulted in some response bias that could have been reduced by a test–retest; however, as it was a convenience sample, this was not possible. Moreover, as both samples differed in their recruitment (a relatively substantial local sample from Tianjin vs. a relatively small national sample from the Netherlands), and used either of the two translated versions of the scale (Cantonese and formal Chinese), this could have contributed to some potential bias in the findings. However, there was no explicit observation or evaluation of the content of the scale that pointed in this direction. This study comprised urban community-dwelling older adults and, therefore, further research is recommended to investigate the DJGLS in other settings such as rural areas or institutionalized older populations. Moreover, it is suggested that the 11-item DJGLS is also translated and validated in Chinese as it would allow additional in-depth comparisons between different communities as well as between the two scales.

Despite the challenging participant recruitment in the Netherlands, this study did manage to include participants from the older Chinese adult community, which is a relatively closed community that is reluctant to participate in surveys. Furthermore, this study is the first, as far as the authors know, to employ a detailed statistical technique such as Kernel Smoothing IRT in addition to the conventional validation methodology to provide novel insights into the cross-cultural equivalence of the DJGLS with respect to items behavior in relation to the degree of loneliness. This study, therefore, broadens the understanding of the cross-cultural equivalence of the Chinese version of the six-item DJGLS. Therefore, it contributes to the importance of the instrument in further research regarding loneliness among older Chinese adults. In conclusion, the findings in this study suggest that the Chinese version of the six-item DJGLS has an adequate and similar item and scalar equivalence for use in Chinese populations. Further research is required to provide additional insights into the cross-cultural equivalence of the scale in native and diasporic Chinese populations.
